# Assessing the mental health literacy of young adults from rural and urban communities in Malawi

**DOI:** 10.1192/bjo.2025.10934

**Published:** 2026-01-09

**Authors:** Beatrice Cynthia Chitalah, Ishani Nanda, Gloria Blessings Chirwa, Joel Limbani Nyali, Sandra Jumbe

**Affiliations:** Department of Research and Corporate Governance, Millennium University, Blantyre, Malawi; Psychology Department, School of Biological and Behavioural Sciences, Queen Mary University of London, London, UK; Centre for Evaluation and Methods, Wolfson Institute of Population Health, Queen Mary University of London, London, UK

**Keywords:** Mental health literacy, young adults, survey, Malawi, rural

## Abstract

**Background:**

Mental health literacy (MHL) is the ability to recognise mental disorders; have knowledge of professional help available, effective self-help and prevention strategies; and have the skills to support others. MHL is linked to better help-seeking behaviours and better management of mental illness. Mental illness prevalence is increasing in Malawi. Assessing MHL in communities crucially helps identify knowledge gaps, informing the development of evidence-based interventions.

**Aims:**

This study assessed the MHL levels of young adults (16–30 years old) in rural and urban communities in Malawi.

**Method:**

A cross-sectional national survey was administered to 682 people across 13 districts in Malawi, using a self-reporting Mental Health Literacy questionnaire (MHLq) that assessed knowledge of mental health problems, erroneous beliefs/stereotypes, first aid skills, help-seeking behaviour and self-help strategies.

**Results:**

Most respondents were either unemployed (36%) or enrolled in school (43%). A total of 73% completed primary or secondary education, and 48% knew someone with a mental illness, but only 14% of this group could specify the illness. The mean MHL score was 111.8 (s.d. 13.9). Individuals with primary and secondary school qualifications had significantly lower scores in factor 2 (erroneous beliefs/stereotypes) and factor 3 (first aid skills and assistance-seeking behaviour) of the MHLq than those with higher education.

**Conclusions:**

This research highlights persisting mental health misconceptions, limited knowledge about specific mental illnesses and low help-seeking behaviour among young adult Malawians. Higher education is linked to a better understanding of mental health. Prioritising community education on causes, signs, treatments and prognosis of mental illness is crucial for increased MHL.

Mental health problems are becoming a significant issue among the youth population globally,^1^ with a 90% treatment gap in South African countries, including Malawi, which does not align with the 10–30% prevalence of common mental disorders among young people alone. Approximately half of adult mental health disorder diagnoses start by 14 years of age, affecting individuals’ functionality and productivity.^2,3^ Untreated mental health problems can have debilitating effects on social life and increase vulnerability to chronic physical conditions.^4^ Research is being conducted to develop preventive approaches to reduce cases and promote mental well-being among young people.^5,6^

Mental health literacy (MHL) is a significant determinant of mental health with the potential of improving health.^7^ It consists of five basic components: recognising mental disorders, knowing available professional help, using effective self-help strategies, skills on supporting others and mental disorders prevention knowledge.^8^ Sufficient MHL helps in understanding risk factors and identifying common mental disorder symptoms, resulting in mental disorder prevention and reduced burden of disease.^9^ It also reduces stigma associated with mental illness, and informs better help-seeking intervention planning, thereby improving treatment-seeking attitudes and adherence among diagnosed cases.

Assessing MHL levels helps identify knowledge gaps and erroneous beliefs about mental health issues, which hinder early recognition and management.^10^ Recognising these issues is crucial for developing interventions aimed at enhancing knowledge and challenging erroneous beliefs, ultimately promoting mental health.^11^

## Study aims

Despite the increasing rates of mental health problems among youth in Malawi, there is limited documentation on programmes targeting MHL.^11,12^ To date, there is no study in Malawi assessing MHL among young adults in its community settings aside from our previous work on the Chichewa-translated MHL questionnaire.^13^ This study therefore aims to report outcomes from our national survey conducted in Malawi, which assessed the MHL levels of young adults in rural and urban communities. Given well-documented disparities in access to health education and mental health services between rural and urban areas,^14,15^ comparing both settings provides essential insight into how context shapes mental health understanding, enhancing the relevance and applicability of findings for developing context-sensitive MHL interventions across Malawi.

## Method

### Procedure

**Fig. 1:**
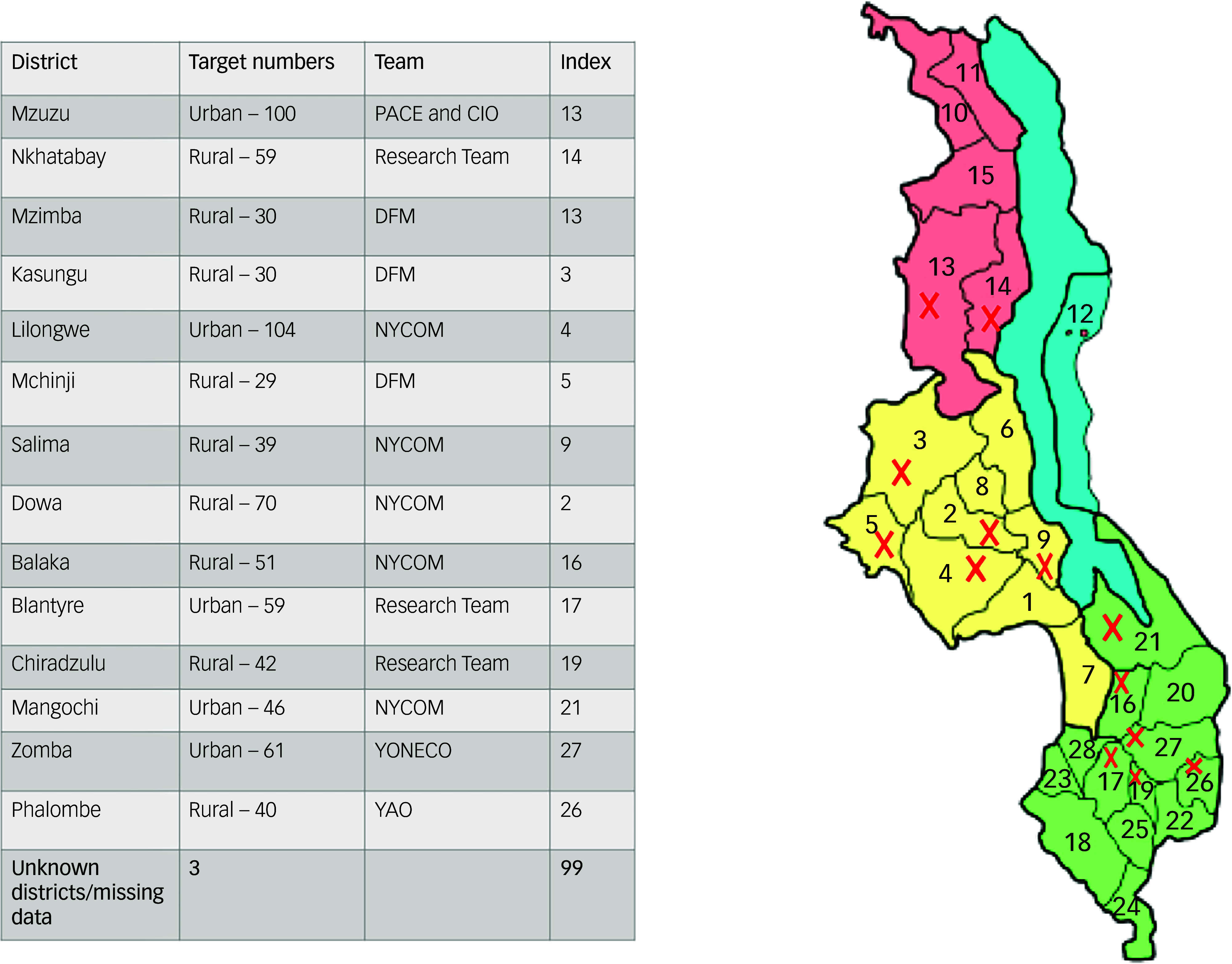
Overview of districts where mental health literacy survey was conducted. PACE, Pace for Social Change; CIO, Citizen Impact Organisation; DFM, Drug Fight Malawi; NYCOM, National Youth Council of Malawi; YONECO, Youth Net and Counselling; YAO, Youth Arms Organisation.

The authors assert that all procedures contributing to this work comply with the ethical standards of the relevant national and institutional committees on human experimentation and with the Helsinki Declaration of 1975, as revised in 2013. The study was approved by the National Committee on Research in the Social Sciences and Humanities on 16 March 2021 before commencing with the current study (reference number P12/20/539). There is no formal guidance on the minimum age at which assent should be sought in Malawi, therefore in practice the age varies between studies and is decided in consultation with local ethics committees.^16^ Considering the low-risk nature of our study and the logistical barrier seeking parental consent may have caused to research participation, the local ethics committee deemed individuals aged 16 years and above as an adult. All research participants (aged 16 years and over) gave their own written consent to participate if they understood what the research involves. A multistage sampling strategy was used, starting with a scoping meeting with our stakeholders Drug Fight Malawi and the National Youth Council of Malawi to identify districts across all three regions of Malawi to aid representative sampling of the general Malawi young adult population (16–30 years). Each district was allocated a quota based on variables such as geographical region, urban versus rural settings, tribal representation and socioeconomic status (see Fig. 1). Surveying was conducted by fieldworkers from several youth-led organisations and the research team (see Fig. 1), between 25 March and 10 December 2021. Fieldworkers randomly approached young people in community hotspots like trading centres and sports grounds to complete a survey. In some districts (Nkhata Bay, Mzuzu, Phalombe), existing stakeholders working as youth workers were used as fieldworkers to better engage with youth and address language issues.

### Sample size

The survey was conducted among 16- to 30-year-old participants who were literate in English or Chichewa. Those unable to read and write independently were excluded.

Slovin’s formula was used to determine an appropriate sample size for this target population.^17^ Our sample size is based on the general population of young adults (16–30 year olds) in Malawi, who make up a quarter of the country’s population, approximately 5 million people. We calculated the sample size by using a 95% confidence interval, 5% margin of error and an s.d. of 0.5. The s.d. value of 0.5 was because of uncertainty regarding how extreme the respondents’ answers would be. This gave a sample size of at least 384. We anticipated a low survey completion/response rate of 50% because an MHL survey study had not been done before, and discussing mental health is culturally sensitive in Malawi. This gave a total sample size of 768.

We used a self-reporting MHL questionnaire (MHLq) for young adults, which assesses four aspects of the MHL construct: knowledge about mental health problems, erroneous beliefs/stereotypes, first aid skills, help-seeking behaviour and self-help strategies.^10^ Psychometric properties of the MHLq regarding validity and reliability have already been shown for this study population.^10^ The questionnaire demonstrated good internal consistency, with Cronbach’s *α* = 0.84 (*n* = 356, aged between 18 and 25 years). We translated the questionnaire into Chichewa (local language) for use in rural communities, and to facilitate its use by mental health practitioners and researchers to screen mental health needs in diverse settings in Malawi.^13^ Preliminary reliability results for this Chichewa MHLq also suggested a reasonably good fit with good overall internal consistency (Cronbach’s *α* = 0.67; *n* = 132), but more psychometric testing with larger samples is needed to further validate the questionnaire for Malawi’s context.^13^ More details regarding the psychometric validity and reliability of this questionnaire for this current sample will be available in a separate forthcoming paper.

### Data collection tool

Data collection followed ethical guidelines, with all participants providing written informed consent before completing the survey. Participants completed part one of the questionnaire, which consisted of questions related to the participant’s gender, age, marital status, nationality, residence, academic qualifications, profession/occupation and proximity to people with mental health problems, including the nature of the relationship (sociodemographics section). Then part two, which contained 29 MHLq items based on the four aforementioned factors, was organised in a five-point Likert response scale (1 = strongly disagree to 5 = strongly agree). For this sample, total scores for the 29 items ranged between 15 and 145. The knowledge of mental health problems factor scores ranged between 7 and 55 (mean score 42.9; s.d. 6.4). Erroneous beliefs/stereotypes factor scores (items 6, 10, 13, 15, 23 and 27 reverse scored) ranged between 0 and 40 (mean score 29.4; s.d. 5.0). The first aid skills and help-seeking behaviour factor ranged between 4 and 30 (mean score 23.0; s.d. 4.8). Factor 4 scores regarding self-help strategies ranged between 4 and 20 (mean score 16.4; s.d. 2.7).

### Data analysis

Descriptive statistics were used to determine sample demographics and levels of missing data, and summarise participants’ MHL levels (Table 1). MHL scores by the four factors (sum of the values of the dimension items) and overall MHL scores (sum of the values of all the items) were computed. Higher values in all dimensions and overall score indicated higher levels of MHL.^10^ The relationship between MHL levels and sociodemographic variables (gender, geographical setting, language and proximity to someone with a mental health problem) was explored by using *t*-tests for independent samples. A one-way analysis of variance (ANOVA) was used to examine the relationship between education levels (primary, secondary and tertiary) and MHL scores. A Pearson’s correlation test was used to assess the relationship between overall MHL score (including four MHLq factor scores) and age.

**Table 1:** Survey respondents’ characteristics

Sociodemographic variables (missing value)	*n*	Valid %
Gender (56)		
Female	336	54
Male	290	46
Mean age, years (s.d.)	21 (3.48)	
Occupation (19)		
Employed full-time	66	9.7
Employed part-time	63	9.2
Unemployed	243	35.6
Student	291	42.6
Marital status (28)		
Single	519	76.1
Married	117	17.2
Divorced	10	1.5
Widowed	8	1.2
Education (33)		
Completed primary	244	35.8
Completed secondary	253	37.1
Higher	152	22.3
Geographical setting (0)		
Urban	282	41.3
Rural	400	58.7
Knowing someone with a mental health problem (8)		
Yes	324	47.5
No	287	42.1
Not sure	60	8.8
Proximity to person with a mental health problem (24)		
Friend	105	30.1
Myself	9	2.6
Relative	59	16.9
Someone else	152	22.3
Language survey completed in		
Chichewa	525	77.0
English	157	23.0

Finally, responses to the open-ended question ‘What type of mental health problem?’ were qualitatively coded for those who ticked ‘Yes’ to the baseline question ‘Do you know anyone who has or had a mental health problem?’. This was done to gauge the types of mental health problems people within participating communities were aware of. Missing data was computed as ‘99’ in the whole data-set. In all hypothesis tests, a level of significance of 5% was considered. All analyses were performed with the statistical analysis program IBM Statistical Package for Social Sciences (SPSS) version 24.0 for Windows.

## Results

A total of 763 participants completed the questionnaire from 13 districts in Malawi (see Fig. 1). Table 1 describes demographic characteristics of the 682 survey participants with complete data, after excluding those with missing ages, those outside the age range of 16–30 years and those who did not respond to any MHLq items (*n* = 7).

Of the young adults, 54% were female and 76% were single. Regarding occupation of respondents, most participants were students (43%), but a significant proportion were unemployed (36%). Most participants’ (73%) highest educational attainment was either primary (36%) or secondary (37%) education. A total of 325 respondents (48%) reported knowing someone who had a mental health problem. Regarding proximity to said person with a mental health problem, 152 of the 325 respondents said it was someone not close to them. The others stated that they had a relative (*n* = 59) or friend (*n* = 105) with a mental health problem. Only nine respondents stated that they themselves had a mental health problem. Finally, 525 out of the 682 respondents (77%) completed the survey in Chichewa language.

### Mental health literacy scores and sociodemographic variables

The mean MHL total score for this sample was 111.8 (s.d. 13.9). Independent *t*-test results showed significant differences in MHLq scores between groups for the sociodemographic variable, language (see Table 2). Those who responded in English had higher total MHLq scores (116.0 ± 12.9) than Chichewa respondents (110.5 ± 14.0, *t*(680) = −4.382, *P* < 0.001), along with all MHLq factors, except for factor 1 (knowledge of mental health problems), where no significant difference was found. However, homogeneity of variances assumption was not met for factors 3 (first aid skills and help-seeking behaviour) and 4 (self-help strategies), indicating substantial differences in variances among groups; therefore, Welch test outcomes are reported instead.

**Table 2:** Differences in mental health literacy (MHLq global score and dimensions) based on language used to complete survey

MHLq factors	Chichewa (*n* = 525) Mean (s.d.)	English (*n* = 157) Mean (s.d.)	*t*-value (d.f. = 680)
MHLq (total score)	110.5 (13.9)	116.0 (12.9)	−4.382***
Knowledge of mental health problems^a^	42.8 (6.5)	43.4 (5.9)	−1.014
Erroneous beliefs/stereotypes	28.8 (4.6)	31.6 (5.3)	−6.447***
First aid skills and help-seeking behaviour^a^	22.7 (5.0)	24.1 (4.0)	−3.841***
Self-help strategies^a^	16.3 (2.8)	16.9 (2.3)	−2.754**

MHLq, Mental Health Literacy questionnaire.

a. Welch test reported as homogeneity of variances assumption was not met for this variable.

** *P* < 0.01; *** *P* < 0.001.

Differences in MHL scores were also noted between participants from rural and urban settings. Rural respondents had significantly higher mean scores than urban participants on the MHLq total score, knowledge of mental health problems, erroneous beliefs/stereotypes and self-help strategies (Table 3). No significant difference in mean scores was noted for factor 3 (first aid skills and help-seeking behaviour) between groups. Interestingly, this was the only factor where rural participants had a mean score that was slightly lower than urban participants. No significant differences in MHLq scores were noted for the other variables (gender and knowledge of someone with mental health problems).

**Table 3:** Differences in mental health literacy (MHLq global score and dimensions) between rural and urban settings

MHLq factors	Rural (*n* = 400) Mean (s.d.)	Urban (*n* = 282) Mean (s.d.)	*t*-value (d.f. = 680)
MHLq (total score)^a^	113.0 (12.7)	110.2 (15.3)	2.494*
Knowledge of mental health problems^a^	43.5 (6.0)	42.2 (6.8)	2.671**
Erroneous beliefs/stereotypes^a^	29.8 (4.5)	29.0 (5.5)	2.041*
First aid skills and help-seeking behaviour	23.0 (4.9)	23.1 (4.6)	−0.230
Self-help strategies^a^	16.7 (2.5)	16.0 (3.0)	3.213***

MHLq, Mental Health Literacy questionnaire.

a. Welch test reported as homogeneity of variances assumption was not met for this variable.

*P < 0.05; ** *P* < 0.01; *** *P* < 0.001.

The relationship between education levels (primary, secondary and tertiary) and MHL scores was examined with one-way ANOVA. However, these failed to meet Levene’s test of homogeneity assumption, meaning there were substantial differences in variances among groups. Therefore, the Welch test (Table 4) was conducted along with the Games–Howell *post hoc* test (Table 5 *)*. There was no statistically significant difference noted between education groups for the total MHL score (*P* = 0.192). Statistically significant differences between education groups were noted for factor 2 (erroneous beliefs and stereotypes) (F(2, 646) = 3.911, *P* = 0.021) and factor 3 (first aid skills and help-seeking behaviour) (F(2, 646) = 7.179, *P* < 0.001).

**Table 4:** Welch robust tests of equality of means

Variable	Statistic^a^	d.f.1	d.f.2	Significance
Factor 1: Knowledge of mental health problems	0.532	2	369.158	0.588
Factor 2: Erroneous beliefs and stereotypes	3.911	2	359.640	0.021
Factor 3: First aid skills and help-seeking behaviour	8.187	2	399.286	<0.001
Factor 4: Self-help strategies	1.613	2	383.862	0.201
Total MHLq score	1.660	2	367.478	0.192

MHLq, Mental Health Literacy questionnaire.

a. Asymptotically *F* distributed.

**Table 5:** Multiple comparisons using the Games–Howell *post hoc* test

Dependent variable	(I) Education grouped	(J) Education grouped	Mean difference (I–-J)	s.e.	Significance	95% Confidence interval	
						Lower bound	Upper bound
Factor 1: Knowledge of mental health problems	Primary education	Secondary education	0.374	0.553	0.777	−0.93	1.67
		Higher education	0.645	0.667	0.598	−0.93	2.22
	Secondary education	Primary education	−0.374	0.553	0.777	−1.67	0.93
		Higher education	0.271	0.702	0.921	−1.38	1.92
	Higher education	Primary education	−0.645	0.667	0.598	−2.22	0.93
		Secondary education	−0.271	0.702	0.921	−1.92	1.38
Factor 2: Erroneous beliefs and stereotypes	Primary education	Secondary education	−0.352	0.413	0.670	−1.32	0.62
		Higher education	−1.561*	0.558	0.015	−2.88	−0.24
	Secondary education	Primary education	0.352	0.413	0.670	−0.62	1.32
		Higher education	−1.209	0.563	0.083	−2.54	0.12
	Higher education	Primary education	1.561*	0.558	0.015	0.24	2.88
		Secondary education	1.209	0.563	0.083	−0.12	2.54
Factor 3: First aid skills and help-seeking behaviour	Primary education	Secondary education	0.468	0.438	0.534	−0.56	1.50
		Higher education	−1.350*	0.452	0.009	−2.41	−0.29
	Secondary education	Primary education	−0.468	0.438	0.534	−1.50	0.56
		Higher education	−1.818*	0.464	<0.001	−2.91	−0.73
	Higher education	Primary education	1.350*	0.452	0.009	0.29	2.41
		Secondary education	1.818*	0.464	<0.001	0.73	2.91
Factor 4: Self-help strategies	Primary education	Secondary education	0.409	0.246	0.221	−0.17	0.99
		Higher education	0.359	0.276	0.394	−0.29	1.01
	Secondary education	Primary education	−0.409	0.246	0.221	−0.99	0.17
		Higher education	−0.050	0.285	0.983	−0.72	0.62
	Higher education	Primary education	−0.359	0.276	0.394	−1.01	0.29
		Secondary education	0.050	0.285	0.983	−0.62	0.72
Total MHLq score	Primary education	Secondary education	0.899	1.196	0.733	−1.91	3.71
		Higher education	−1.906	1.478	0.402	−5.39	1.58
	Secondary education	Primary education	−0.899	1.196	0.733	−3.71	1.91
		Higher education	−2.805	1.539	0.164	−6.43	0.82
	Higher education	Primary education	1.906	1.478	0.402	−1.58	5.39
		Secondary education	2.805	1.539	0.164	−0.82	6.43

MHLq, Mental Health Literacy questionnaire.

*The mean difference is significant at the 0.05 level.

Regarding factor 2, the Games–Howell *post hoc* test revealed that MHL factor 2 scores were statistically significantly lower for those who had completed primary school (29.0 ± 4.5) compared with those who had completed higher education (30.6 ± 5.9). There was no statistically significant difference between the primary and secondary groups (*P* = 0.670), or the secondary and higher education groups (*P* = 0.083). Similarly, for factor 3, there was no statistically significant difference between the primary and secondary groups (*P* = 0.534). However, MHL factor 3 scores were statistically significantly lower for those who had completed primary school (22.9 ± 4.7, *P* = 0.009) and secondary school (22.5 ± 5.1, *P* < 0.001) compared with those in higher education (24.3 ± 4.2).

Taken together, these results suggest that education has an effect on MHL scores. Specifically, our results suggest that those who go on to study at university or college or have higher education qualifications have a better understanding of mental health than those with primary or secondary school qualifications. It should be noted that there does not seem to be much difference in MHL between those who have completed primary and secondary education.

A Pearson’s correlation test was used to assess the linear relationship between MHL score (including four MHLq factor scores) and age (Table 6). There was a significant but very weak negative relationship between the MHL total score and age variables (*r*(680) = −0.088, *P* = 0.022). In terms of the MHLq factor scores, there was also a significant negative relationship between the factor 2 (erroneous beliefs and stereotypes) scores and age (*r*(680) = −0.083, *P* = 0.029). No significant relationship was noted with the other 3 MHLq factors. Overall results suggest that younger adults may have higher MHL than older adults.

**Table 6:** Correlations between mental health literacy scores and age

MHLq factors		Age
Factor 1: Knowledge of mental health problems	Pearson correlation	−0.062
	Significance (two-tailed)	0.105
	*n*	682
Factor 2: Erroneous beliefs and stereotypes	Pearson correlation	−0.083*
	Significance (two-tailed)	0.029
	*n*	682
Factor 3: First aid skills and help-seeking behaviour	Pearson correlation	−0.045
	Significance (two-tailed)	0.237
	*n*	682
Factor 4: Self-help strategies	Pearson correlation	−0.071
	Significance (two-tailed)	0.062
	*n*	682
Total MHLq score	Pearson correlation	−0.088*
	Significance (two-tailed)	0.022
	*n*	682

MHLq, Mental Health Literacy questionnaire.

*Correlation is significant at the 0.05 level (2-tailed).

### Types of mental health problems

Out of the 325 individuals who disclosed knowing someone with an mental health problem, only 14.1% were able to identify what the specific mental health problem of the person they knew was. In particular, they reported knowing someone with conditions such as post-traumatic stress disorder, anxiety, stress, schizophrenia and bipolar disorder (Table 7); 34.2% wrote terms like mental disorder, brain problem (*vuto la ubongo* and *mutu sugwira ntchito* are the Chichewa equivalents) or madness (*misala*, *kupenga* and *kuzungulira mutu* are the Chichewa equivalents), without being exact on the type of mental disorder. Seven individuals mentioned epilepsy, a neurological condition. The rest of the participants (31%) were unable to identify specific mental health problems or disorders; instead, they mentioned potential signs and symptoms (e.g. wandering about aimlessly, talking to self) or antisocial behaviours (e.g. stealing, fighting).

**Table 7:** Types of mental health problems reported by respondents

Type of mental health problem (Chichewa and English responses combined)	Frequency
Depression/*kukhumudwa*/etc.	30
Madness/*misala*/*kupenga*/mental disorder	104
Anxiety/*nkhawa*/etc.	8
Stress	7
Post-traumatic stress disorder	2
Brain problems/*vuto la ubongo/mutu sugwira ntchito*	17
Epilepsy	17
Substance use problems	10

## Discussion

Many people have a higher chance of developing mental health disorders, because of the global increase in mental health illnesses during a lifetime.^8^ This study aimed to assess MHL levels among young adults in Malawi, focusing on rural and urban settings. Just over half of our sample were women, 76% were single and a third were unemployed. Most participants completed primary or secondary education. The sample was representative in terms of gender, as women make up 52% of the total Malawi population and education status. However, unemployment in our sample was higher than the national estimate of 9%.^18^

The survey revealed that 48% of respondents knew someone with mental health issues, but only 14% could identify the specific disorder. This lack of identification can lead to delayed treatment and intervention, reduced quality of life and increased burden on families and caregivers.^19–21^ The study also found significant differences in mental health literacy levels between education groups for ‘erroneous ideas and stereotypes’ (F(2, 646) = 3.911, *P* = 0.021) and ‘first aid skills and help-seeking behaviour’ (F(2, 646) = 7.179, *P* = 0.001). As expected, the higher one’s education level, the higher one’s MHL level. There was, however, no significant difference in MHL scores between primary and secondary education participants. Higher education levels resulted in a lower likelihood of supporting negative ideas or stigmatising notions.^22^

The finding of rural respondents scoring higher total MHL scores than urban counterparts is interesting and somewhat counterintuitive, as higher (health) literacy is often associated with urban areas because of the assumed better access to education and services.^14^ However, it is worth noting that 82% of Malawi’s population live in rural areas,^18^ making the country a predominantly rural country. This rural overrepresentation likely heavily influenced the overall mean, possibly masking or diluting urban scores. There are several other plausible explanations to consider regarding this result. All rural respondents used the Chichewa MHLq; perhaps those reading the questionnaire in the local language interpreted questions more accurately, improving their scores. Third, Malawi’s rural areas often benefit from targeted non-governmental organisation initiatives and active community health worker programmes, where health surveillance assistants conduct mental health education and screenings in villages, which may boost awareness and literacy among rural residents.^23^ We did also include informal settlements (e.g. Ndirande) and peri-urban locations within the urban areas, so it is possible that the urban sample included individuals exposed to the extremes of very low and very high quality of education alongside extreme socioeconomic backgrounds, diluting average scores. Further, homogeneously educated rural youth (e.g. primarily Chichewa-educated secondary school-leavers) may perform more consistently well. Finally, although urban environments offer more formal mental-health services (which in Malawi is still very limited), rural communities rely more on communal support systems like family, village elders and faith-based organisations, to manage mental health issues.^24^ This everyday ‘familiarity through exposure’ could enhance rural participants’ practical mental-health literacy – even if only implicitly.^25^

Another key thing to acknowledge is how cultural beliefs impact mental health in Malawi. Socio-cultural supernatural explanations for mental illnesses remain common and coexist with biomedical ones. Large community and clinic surveys in Malawi report that many people attribute mental illness to spirits, witchcraft, curses or God’s punishment, and strongly influence first-line help-seeking.^26^ Where problems are often framed as spiritual or moral, people frequently seek initial care from traditional healers or religious leaders, typically delaying or substituting biomedical care. Recent Malawian and regional reviews document this pathway and call for respectful collaboration between sectors to improve mental healthcare.^24^ Chilale et al suggest reinforcing a biopsychosocial intervention paradigm for mental disorder treatment that incorporates socio-cultural explanations can increase help-seeking behaviour.^21^ Very recent research from Malawi shows persisting high levels of stigma (including internalised stigma and discriminatory treatment) toward people with mental illnesses.^27^ This stigma is culturally specific and shaped by previously mentioned local beliefs about causation and morality. There is a critical need for anti-stigma teaching initiatives in Sub-Saharan Africa, where societal separation and stigmatisation of mental illness is prevalent. Incorporating such initiatives into mental health policies is also crucial.^28^

This study has several strengths. This study is the first in Malawi to assess mental health literacy among young adults in community settings. With a large sample size of 682 participants, the findings are generalisable to the young adult population aged 16–30 years in Malawi. The sample representativeness was well-considered because of a sampling strategy accounting for key variables, including participants from both urban and rural areas across the country. The open-ended questions in the survey provided crucial depth to the data. The cross-sectional survey approach was cost-effective and efficient. Having both English and Chichewa versions of the MHLq made the survey accessible to a wider range of individuals in both rural and urban communities.

Study limitations include its focus on young adults (16–30 years old), which limits its applicability to different ages in the community. We had to exclude 81 respondents from our analyses because of missing data. Lack of resources and time meant we could not recoup this lost data. In future, we will need to have a strategic protocol in place with fieldworkers to minimise missing data. Finally, the cross-sectional survey data has limited ability to draw causal inferences because variables were measured at a single point in time.^29^ The study provides only a snapshot of associations and cannot capture changes or temporal relationships.

To conclude, the study aimed to assess mental health knowledge and attitudes among young adults, highlighting gaps in mental health education. The survey highlights the need for educational interventions targeting MHL at a younger age, particularly in primary and secondary schools, to reduce stereotyping and negative/stigmatising beliefs toward individuals with mental health problems, enabling them to seek professional help more effectively. Community education on mental health should focus on underlying factors, symptoms, treatment and prognosis. Schools should provide mental health education with treatment and aid options to reduce stereotypes.^20^ Research shows that providing an MHL education curriculum improves student knowledge of mental illness, reduces stigmatising attitudes and makes it easier to seek assistance and identify problems early. Alongside our own recent work,^30^ there is growing evidence in global mental health research supporting the effectiveness of school-based mental health literacy programmes in low-resource settings.^31,32^ This approach increases the success rate of treatments,^33^ as individuals who can identify their own or family members’ mental health issues may receive prompt help and treatment.^8,21^ Embedding MHL in teacher training, secondary schools and universities to improve mental health knowledge and address stigma at scale can also be a cost-effective, sustainable approach that supports Malawi’s existing under-resourced mental healthcare system, particularly the lack of youth-specific services^12^ and the scarcity of mental health professionals.^14^

This survey data can be used by mental health practitioners and academics to promote MHL in rural and urban settings, especially among disadvantaged groups. Adequate MHL is crucial for early detection and treatment of mental health issues and promoting mental wellness. However, there is limited research on MHL in low-income countries like Malawi.^12^ More longitudinal studies are needed to understand its changes over time and to translate data collection tools into native languages. Culturally relevant MHL programmes should be delivered across communities, considering individual literacy levels.

## Data Availability

The data that supports the findings of this study is available on request from the corresponding author, S.J.
